# Beliefs About the Use of Herbs Before and After COVID-19: A Cross-Sectional Study in Saudi Arabia

**DOI:** 10.7759/cureus.49624

**Published:** 2023-11-29

**Authors:** Yosra Z Alhindi, Aeshah A Habib, Reham H Alhusini, Afnan S Bahha, Afnan E Alqurashi, Raghad S Almatani, Bashayer Alharbi, Mayam H Alharthy, Farah R Alharbi, Orjwan Sendi, Raghad Alamoudi, Nouf A Hijji, Tala S Alkhayat

**Affiliations:** 1 Pharmacology and Toxicology, Faculty of Medicine, Umm Al-Qura University, Makkah, SAU; 2 Pharmacy, Umm Al-Qura University, Makkah, SAU; 3 Pharmacy, Umm Al-Qura University, Makkkah, SAU

**Keywords:** saudi arabia, cross-sectional, covid-19, herbs, beliefs

## Abstract

Background: The COVID-19 pandemic was declared a public health emergency of international concern by the World Health Organization on March 12, 2020. Natural products and herbal medicine have been used since ancient times to relieve and treat disorders and infections, as well as increase immunity. Despite the beneficial effects of herbal medications, there are many side effects or interactions with other medications or foods that might occur.

Aim: This study aims to explore the beliefs of Saudi people towards the use of herbal medicine for COVID-19 infection.

Method: A cross-sectional study using an online survey was conducted in Saudi Arabia between January 2021 and January 2023. This survey was generated based on a deep review of the literature on COVID-19 as well as the use of medicine and herbal medicine to treat this infection. SPSS software was used to analyze the data, with a significance level of p < 0.05.

Results: A total of 1,230 individuals participated in this study. More than half (67.6%, n = 831) were females. Around 32.4% (n = 399) of the participants were aged 20-40 years. The majority were married (77.2%, n = 947), Saudi (96.5%, n = 1186), and living in central provinces (62.5%, n = 768) of the Kingdom. More than half of them (70.0%, n = 861) were bachelor's degree holders; 42.3% and 2.4% (n = 29) reported that they had been or were currently infected with COVID-19. Around 33.0% (n = 405) of the participants reported that they had used herbal products or nutritional supplements during the pandemic period to protect themselves from the disease.

Conclusion: Our study showed that Saudi Arabia's public takes dietary supplements or herbal products to fight against the illness. We recommend that the Ministry of Health conduct more educational efforts to raise public awareness about disease transmission pathways and preventive actions. Furthermore, to guarantee patient safety, the use of herbal products should be supported by a professional counselor.

## Introduction

The COVID-19 pandemic was declared a public health emergency of international concern by the World Health Organisation on March 12, 2020 [1]. COVID-19 is characterised by a very high transmission property that can cause severe damage to patients' lungs [1]. In Saudi Arabia, the first case of COVID-19 was reported in March 2020, with a high spreading rate throughout the country [2]. The Saudi government started restriction measures for the educational and transportation systems [3]. However, the virus was still spreading, with more than 145 thousand cases in the kingdom [4].

Indeed, there is no definitive treatment for COVID-19, which has raised a flag of concern globally. Thus, there was a good amount of attention towards seeking other ways than using drugs or medicine to treat this infection and increase immunity against it [5].

Natural products and herbal medicine such as mint, oregano oil, and garlic were used in ancient history to relieve and treat disorders and infections, as well as increase immunity [5-7]. Despite the beneficial effects of herbal medications, there are many side effects or interactions with other medications or foods that might occur [8].

Nowadays, there has been a huge increase in the use of herbal medicine by people who are afraid of COVID-19 without a prescription or reviewing their situation with an expert [9,10]. In addition to enhancing well-being, optimal nutrition may help reduce the risk and morbidity of COVID-19, which is brought on by the severe acute respiratory syndrome coronavirus 2 (SARS-CoV-2) virus [9]. Studies showed recommendations emphasised the need for vitamins, minerals, and antioxidants such as zinc and vitamins C, A, and D to keep the immune system functioning properly [10]. There is no evidence to suggest that dietary supplements can prevent COVID-19. Nonetheless, it was noted that supplementing with zinc, selenium, and vitamins C and D may be advantageous for people who have respiratory virus infections, are susceptible to them, or have nutrient deficiencies [11].

Our main aim of this study was to explore the beliefs of Saudi people towards the use of herbal medicine for COVID-19 infection.

## Materials and methods

Study design and study population

This study is a cross-sectional study using an online cross-sectional survey (Google Form) that was conducted in Saudi Arabia between January 2021 and January 2023. This survey was generated based on a deep review of the literature on COVID-19 as well as the use of medicine and herbal medicine to treat this infection [12-14].

In addition, three researchers examined the questionnaire instrument to assess the questions' adequacy, appropriateness, relevancy, clarity, and validity. Validation was done by a group of people who answered the survey and were unrelated to our results. In addition to covering sociodemographic information, the survey delves into people's views regarding the use, sources of information, and availability of herbal products and food supplements, as well as their perceived ability to fend off the pandemic.

Sampling strategy

Through social media, a convenient sample of qualified participants was recruited to participate in the research (Facebook, Twitter, Snapchat, Instagram, and WhatsApp). Since every subject gave their free will to the research, they were all excluded from the need for formal, informed consent. The survey began with a clear explanation of the study's goals and objectives.

Participants who are Saudi Arabian citizens and are at least 18 years old were included. Participants were not allowed to participate if they could not understand Arabic or were younger than 18 years old.

Sample size

We used the strategy of a confidence interval of 95%, a standard deviation of 0.5, and a margin of error of 5%; the required sample size was 380 participants. However, we collected 1230 in case of any missing data.

Statistical analysis

SPSS software, version 25 (IBM Corp., Armonk, NY, USA), was used to analyze the data. While frequencies and percentages were used to report categorical variables, the mean standard deviation was used to report continuous variables.

Ethical considerations

This study was approved by the Umm Al-Qura IRB Committee, Approval Number: HAPO-02-K-012-2023-11-1851.

## Results

Study participants basic characteristics

The total number of subjects in our study was 1,230. Females were 67.6% (n = 831). Subjects aged between 20 and 40 years were about 32.4% (n = 399). Married participants were (77.2%, n = 947), Saudi (96.5%, n = 1186), and lived in the centre of Saudi Arabia (62.5%, n = 768). Moreover, 70.0% (n = 861) of them hold bachelor degrees, and 42.3% (n = 520) of them are working. In addition, 31.5% (n = 388) at least know a member of their family has a career in health. About 15% (n = 185) of our study subjects have a history of chronic disorders such as diabetes or hypertension. Moreover, 2.4% (n = 29) admitted having a history of COVID-19 infection (Table 1).

**Table 1:** Basic characteristics of study participants % Data presented in N

Characteristics	Frequency (%)
Gender	
Female	831 (67.6)
Age categories (year)	
18–24	143 (11.6)
25–39	256 (20.8)
40–59	200 (2.4)
60 and above	155 (1.9)
Marital status	
Single	283 (22.8)
Married	947 (77.2)
Nationality	
Saudi	1186 (96.5)
Non-Saudi	44 (3.5)
Residence area	
Central province	768 (62.5)
Eastern province	112 (9.1)
Western province	45 (3.6)
Northern province	55 (4.4)
Southern province	250 (20.3)
Education level	
Completed primary education	366(29.7)
Bachelor's degree	520 (42.3)
Higher education	344 (28.0)
Employment status	
Student	112 (9.10)
Healthcare-related career	388 (31.5)
Employee	520 (42.3)
Unemployed (retired or housewife)	210 (17.7)
Chronic disease history	
Yes	185 (15)
Where infected/currently infected with COVID-19?	
Yes	29 (2.4)

Views about the application of dietary supplements and herbal goods as a preventative measure

Approximately 33.0% (n = 405) of the participants stated that they took dietary supplements or herbal products to ward off illness during the pandemic. 10.2% (n = 125) of the respondents also mentioned that they had used these products for a period before stopping. But 12% (n = 147) of people are still utilising it.

The participants were mostly motivated to test herbal products by social media and the Internet (55.6%, n = 683), and 33.4% (n = 411) of them stated that they bought herbal items from stores. When asked what kinds of dietary supplements they had taken or were taking at the time, 90.3% (n = 1110) said they were taking vitamin C.

Regarding the usage of food supplements and/or herbal products as a preventive approach, 14.6% of participants think that consuming garlic boosts immunity and lowers the risk of getting COVID-19. Vitamin C was regarded by 34.1% of the participants as having a function in treating or lowering the risk of getting COVID-19, which was followed by the belief in herbal products (Tables 2-3).

**Table 2:** Opinions about the application of dietary supplements and herbal goods as a preventative measure % Data presented as N

Variable	Frequency (%)
Using herbs or supplements during the pandemic time (n = 1230)	
Yes	405 (33.0)
No	825 (67.0)
I used it for a while and then stopped it	125 (10.2)
Using herbs or supplements after the pandemic time (n = 1230)	
Yes	147 (12.0)
No	1083 (88.0)
If yes, who suggested you take a nutritional/herbal supplement? (n = 1230)	
Dietician/physician/pharmacist/nurse/health practitioner	200 (16.2)
Social media and other websites	683 (55.6)
Recommendation from friend or relative	347 (28.2)
If yes, where did you get it?	
Herbal product shop	410 (33.4)
Pharmacy	211 (17.2)
Home	332 (27.0)
Internet	277 (22.4)
What dietary supplement did you use before/use now during the pandemic? (You can choose more than one answer) (n = 1230)	
Vitamin C	1110 (90.3)
Royal Jelly	120 (9.7)

**Table 3:** Views about the application of dietary supplements and herbal goods as a preventative measure % Data presented as N

Views about the application of dietary supplements and herbal goods as a preventative measure	Yes	No	Maybe	I don’t know
Drink turmeric tea to elevate the immune system immunity and to decrease the chance of infection with COVID-19	135 (11.0%)	274 (22.3%)	286 (23.3%)	533 (43.4%)
Ginger tea helps to elevate the immune system immunity and to decrease the chance of infection with COVID-19	423 (34.4%)	410 (33.4%)	393 (32.0%)	4 (0.02%)
Garlic helps to elevate the immune system immunity and to decrease the chance of infecting with COVID-19	180 (%14.6)	185 (15.0%)	412 (33.5%)	453 (36.9%)
Eating onions helps to elevate the immune system immunity and to decrease the chance of infecting with COVID-19	253 (20.6%)	247 (20.1%)	354 (28.8%)	375 (30.5%)
Fish oil or Omega-3 helps to elevate the immune system immunity and to decrease the chance of infection with COVID-19	116 (9.5%)	243 (19.8%)	314 (25.6%)	554 (45.1%)
Ginseng extract helps to elevate the immune system immunity and to decrease the chance of infection with COVID-19	43 (3.4%)	257 (20.9%)	202 (16.5%)	728 (59.2%)
Vitamin C helps to elevate the immune system immunity and to decrease the chance of infection with COVID-19	360 (29.3%)	168 (13.7%)	410 (33.4%)	289 (23.5%)
Vinegar helps to elevate the immune system immunity and to decrease the chance of infection with COVID-19	73 (5.9%)	360 (29.3%)	230 (18.7%)	567 (46.1%)
Salted water helps to elevate the immune system immunity and to decrease the chance of infection with COVID-19	123 (10.0%)	410 (33.4%)	263 (21.4%)	432 (35.2%)
Sesame oil helps to elevate the immune system immunity and to decrease the chance of infection with COVID-19	15 (1.2%)	634 (51.6%)	85 (6.9%)	495 (40.3%)
Nutritional supplements help to elevate the immune system immunity and decrease the chance of infection with COVID-19	193 (15.7%)	290 (23.6%)	390 (32.2%)	350 (28.5%)
Vitamin and herbal supplements help to elevate the immune system immunity and to decrease the chance of infection with COVID-19	54 (4.4%)	704 (57.3%)	172 (14.0%)	299 (24.3%)

## Discussion

This study investigated the beliefs about using herbal medicine, products, and food during and after the COVID-19 pandemic in Saudi Arabia. Our finding revealed the importance of educating the general population of the community about the disease itself and how to use herbs under the supervision of a physician, pharmacist, or specialist, especially with subjects who suffer from chronic diseases that can affect other medications they are using, in addition to knowing how to use herbs effectively to prevent and protect them from the virus or any transmission.

Indeed, the transmission of COVID-19 holds global concern as it is characterised by very fast and dangerous transmission [15]. According to available data, the COVID-19 causative virus spreads by respiratory droplets or by contaminated hands encountering the mucosa of the mouth, nose, or eyes. Contaminated hands can also spread the virus from one surface to another, which promotes indirect contact transmission. To stop the pandemic from spreading, the health authorities are taking numerous preventive measures into consideration due to these facts and the lack of viable vaccines or treatments [16,17].

Based on lessons learned from the 2012 Middle East Respiratory Syndrome (MERS) epidemic, the Ministry of Health in the Kingdom of Saudi Arabia promptly launched several campaigns, introduced mobile health applications and call centres, and informed the public about COVID-19 and related topics to raise public awareness, encourage active learning, and encourage the public to look up information about relevant preventive measures such as the protocol of washing hands and the benefits of staying at home [18,19].

Approximately 33.0% (n = 405) of the participants in our survey stated that they had used and 12.0% (n = 174) are now utilising dietary supplements or herbal medicines to protect themselves from the COVID-19 pandemic. Worldwide, people use herbal items and dietary supplements to improve their health or treat conditions related to their health. Medicinal plants are being considered as a possible source of effective treatment for sickness due to the lack of novel and effective pharmaceutical treatments to fight drug-resistant infections and viral diseases. Because there is currently no effective COVID-19 vaccine or treatment, the rapid spread of the newly discovered pandemic has increased public anxiety, terror, and rage [20]. As could be predicted, people who are aware of the risks associated with these infections seek out self-care strategies and use non-traditional treatments like food supplements and herbal products such as garlic, oregano, mint, and beetroots to lower their risk and gain more control over their condition [21-23].

Herbal products such as garlic, mint, and oregano oil are increasingly being used by patients, and a large body of research has documented their application in the treatment of serious emerging infectious diseases, including SARS and MERS [24,25]. The general public's use of herbal remedies and supplements as a prophylactic precaution during the COVID-19 pandemic in the Middle East has not been extensively studied. According to a recent Moroccan study, during the COVID-19 epidemic, 23 distinct types of therapeutic plants from 11 different botanical families were used. The Lamiaceae, Cupressaceae, and Zingiberaceae families were the most significant. According to EL Alamlab et al. [9], the most often utilised plants were garlic, olive, onion, and ginger [26].

The public frequently uses ginger, onion, and garlic, and there is a widespread assumption that these foods can boost immunity and lower the risk of contracting COVID-19 [27]. Our investigation supported these findings. According to earlier research, there is a widespread perception that herbal products are high-quality and safer than prescription drugs. Herbal medications and supplements were frequently utilised for reasons such as family customs and habits, positive experiences with herbal medicine in the past, and dissatisfaction with conventional therapy [28]. Because it supports the diverse cellular processes of the innate and adaptive immune systems, vitamin C is one of the most widely used vitamins in a variety of populations [27,29]. Furthermore, vitamin C supports the function of the epithelial barrier, encourages oxidant scavenging activity, builds up in phagocytic cells (like neutrophils), and stimulates a few defence mechanisms that ultimately aid in the destruction of microorganisms [30,31]. Garlic supplements made of natural goods are also widely used by the public, which is thought to be related to their antioxidant, antibacterial, and anti-inflammatory properties that help safeguard human health [32].

The fact that a good sample of individuals was gathered during this crucial time is what makes our study strong. There are certain limitations, though. The cross-sectional survey methodology of the study made it more difficult for us to determine if study variables were causally related. The evaluation of people's knowledge regarding the COVID-19 pandemic and the use of herbal products and food supplements globally and in the Middle East has not been extensively studied, which made it difficult to compare our results with those of Arabic-speaking nations with comparable socioeconomic status and cultural norms. Furthermore, we may not have reached all the intended population and may have overlooked some vulnerable populations that were not reachable due to the use of an online survey for data collection.

## Conclusions

Our study's conclusions showed that Saudi Arabia's public is good at taking dietary supplements or herbal products to fight against the illness. We recommend that the Ministry of Health conduct more educational efforts to raise public awareness of disease transmission pathways and preventive actions. Furthermore, to guarantee patient safety, the use of herbal products should be supported by a professional counsellor, for example, by an electronic-supported system that is reviewed regularly.
